# Web Alert: *Bacillus* in microbial biotechnology: An annotated selection of World Wide Web sites relevant to the topics in microbial biotechnology

**DOI:** 10.1111/1751-7915.13996

**Published:** 2021-12-31

**Authors:** Lawrence P. Wackett

**Affiliations:** ^1^ Department of Biochemistry Molecular Biology & Biophysics, BioTechnology Institute University of Minnesota St. Paul MN 55108 USA

## *Bacillus* spores in biotechnology

### https://journals.asm.org/doi/10.1128/AEM.01096‐20

This review explores innovative applications of *Bacillus cereus* spores in biotechnology.

## *Bacillus* cell factories

### https://microbialcellfactories.biomedcentral.com/articles/10.1186/s12934‐020‐01436‐8

This represents a very broad review of the many applications of *Bacillus subtilis* in industrial biotechnology.

## Growing *Bacillus*

### https://www.sensientbionutrients.com/post/growing‐bacillus‐can‐be‐fun‐introduction/

Sensient bionutrients provides media additives for growing microbes, including *Bacillus* strains, for the biotechnology industries. This blog provides a simple but useful discussion of *Bacillus* growth.

## Bacillus thuringiensis pesticide

### http://npic.orst.edu/factsheets/btgen.html

This page is connected to the National Pesticide Information Center. It treats *Bacillus thuringiensis* as one of many pesticides, discussing how it has been evaluated for toxicological safety.

## Bacillus thuringiensis

### https://www.sciencedirect.com/topics/biochemistry‐genetics‐and‐molecular‐biology/bacillus‐thuringiensis

This ScienceDirect page is a compilation of different resources on *Bacillus thuringiensis*, a naturally occurring soil bacterium that produces an insecticidal protein. Different pesticidal proteins in different *B. thuringiensis* strains show toxic effects on specific insects, making this as a very useful set of biopesticides.

## *Bacillus* spp. and their enzymes

### https://www.frontiersin.org/articles/10.3389/fmicb.2020.01782/full

This review largely focuses on *Bacillus* strains as a native source of enzymes and as expression systems for making a variety of enzymes of industrial importance.

## Extracellular protein production by *Bacillus* strains

### https://microbialcellfactories.biomedcentral.com/articles/10.1186/s12934‐017‐0649‐1

Industrial *Bacillus subtilis* strains have many mutations to make them more effective in recombinant protein production. This paper discusses a number of these types of mutations.

## Bacillus licheniformis risk assessment

### https://www.epa.gov/sites/default/files/2015‐09/documents/fra005.pdf

This site contains an early risk assessment of *Bacillus licheniformis* by the U.S. Environmental Protection Agency relevant to its assignment of the organism to be Generally Regarded As Safe (GRAS).

## *Bacillus* patents in agricultural applications

### https://link.springer.com/chapter/10.1007/978‐981‐13‐7466‐1_8

This article is within a journal on intellectual property in microbiology than focuses on agriculture. *Bacillus thuriengiensis* biopesticides and natural product producing *Bacillus* strains figure prominently in this issue.

## Generally Regarded As Safe (GRAS): *Bacillus* and others

### https://www.liebertpub.com/doi/10.1089/ind.2016.0011

Industrial enzymes are produced in microorganisms that are Generally Regarded As Safe (GRAS) to preclude any problems in the event that any fragments of the organism contaminate the product enzyme.

## SporeGen

### http://sporegen.com/

SporeGen is a company that focuses on *Bacillus* for vaccines, novel foods, drug delivery and strain development and assessment.

## *Bacillus* laundry washing enzymes

### https://scialert.net/fulltext/?doi=jm.2008.661.672

*Bacillus* enzymes are important in laundry washing powders. A major enzyme class is proteases, which are essential to lift protein materials out of cloth.

## Thermostable *Bacillus* alpha‐amylase

### https://www.megazyme.com/alpha‐amylase‐thermostable‐bacillus‐sp

This page is a specification sheet for a commercial alpha‐amylase from a *Bacillus* sp. Alpha‐amylases are used in decomposing starch to use the released sugars for commercial purposes.

## Bacillus licheniformis used for chickens

### https://www.frontiersin.org/articles/10.3389/fmicb.2021.623739/full

This study examined feeding *B. licheniformis* to chickens as an alternative to an antibiotic, for preventing intestinal disease.

## *Bacillus* and natto

### https://journals.asm.org/doi/10.1128/AEM.00448‐11

Some *Bacillus* strains are known to produce extracellular poly‐*gamma*‐glutamic acid, and that is applied to the production of natto, a fermented soybean food.

## Vitamin B_12_ production

### https://www.frontiersin.org/articles/10.3389/fbioe.2020.570828/full

This article describes the production of vitamin B_12_ by *Bacillus subtilis* and other bacteria.

